# Solute carriers affect *Anopheles stephensi* survival and *Plasmodium berghei* infection in the salivary glands

**DOI:** 10.1038/s41598-017-06317-6

**Published:** 2017-07-21

**Authors:** J. Couto, S. Antunes, R. Pinheiro-Silva, V. do Rosário, J. de la Fuente, A. Domingos

**Affiliations:** 1 Instituto de Higiene e Medicina Tropical, Universidade Nova de Lisboa (GHMT-IHMT-UNL), Rua da Junqueira 100, 1349-008 Lisboa, Portugal; 2 Global Health and Tropical Medicine - Instituto de Higiene e Medicina, Universidade Nova de Lisboa (GHMT-IHMT-UNL), Rua da Junqueira 100, 1349-008 Lisboa, Portugal; 3 SaBio. Instituto de Investigación en Recursos Cinegéticos IREC, CSIC-UCLM-JCCM, Ronda de Toledo s/n, 13005 Ciudad Real, Spain; 4 Department of Veterinary Pathobiology, Center for Veterinary Health Sciences, Oklahoma State University, Stillwater, OK 74078 USA

## Abstract

Malaria is caused by mosquito-borne *Plasmodium* spp. parasites that must infect and survive within mosquito salivary glands (SGs) prior to host transmission. Recent advances in transcriptomics and the complete genome sequencing of mosquito vectors have increased our knowledge of the SG genes and proteins involved in pathogen infection and transmission. Membrane solute carriers are key proteins involved in drug transport and are useful in the development of new interventions for transmission blocking. Herein, we applied transcriptomics analysis to compare SGs mRNA levels in *Anopheles stephensi* fed on non-infected and *P. berghei*-infected mice. The *A. stephensi* solute carriers *prestinA* and *NDAE1* were up-regulated in response to infection. These molecules are predicted to interact with each other, and are reportedly involved in the maintenance of cell homeostasis. To further evaluate their functions in mosquito survival and parasite infection, these genes were knocked down by RNA interference. Knockdown of *prestinA* and *NDAE1* resulted in reduction of the number of sporozoites in mosquito SGs. Moreover, *NDAE1* knockdown strongly impacted mosquito survival, resulting in the death of half of the treated mosquitoes. Overall, our findings indicate the importance of prestinA and NDAE1 in interactions between mosquito SGs and *Plasmodium*, and suggest the need for further research.

## Introduction

Over the last 15 years, massive prevention measures and new treatment tools have greatly decreased the global malaria burden. However, despite these advances, about 214 million new malaria cases and 438,000 associated deaths were registered in 2015 alone^1^. Malaria is caused by mosquito-borne *Plasmodium* parasites. In particular, adult female mosquitoes of the genus *Anopheles* are efficient *Plasmodium* vectors in many diverse ecosystems^2, 3^. *Anopheles stephensi* is a competent vector for both *Plasmodium falciparum* and *P. vivax*, the most virulent malaria-associated species^3^, as well as for *Plasmodium* species that infect rodents and, thus, this mosquito species is widely used as a laboratory model. *A. stephensi* is found throughout the Indian subcontinent—with its territory extending from the Arabian Peninsula, Iran, and Iraq, to Bangladesh, southern China, Myanmar, and Thailand^4^.

Malaria transmission from mosquitoes to vertebrate hosts occurs through the mosquito’s salivary glands (SGs), and for this reason many studies have focused on this organ to develop new malaria control measures^5–9^. Sporozoite invasion of mosquito SGs is receptor-mediated, and involves several parasite and SG proteins^10, 11^. Characterization of the salivary components involved in parasite infection and transmission would greatly enhance our understanding of the mosquito–host–pathogen interface, and potentially reveal candidate targets for malaria prevention and control.

Sialotranscriptomic and functional genomic studies of *Anopheles* spp. after feeding or infection have created transcriptome catalogues that reveals the influence of *Plasmodium* infection process in certain genes^5, 12^. Recently, a role of a membrane glucose transporter gene in *Anopheles gambiae* was reported in malaria transmission using RNA interference studies^5^. Membrane proteins are responsible for the transport of a high diversity of substrates through the SGs, maintaining basic cell physiological functions and preserving optimal conditions for completion of the parasite life cycle^5, 13, 14^.

Solute carrier (SLC) families include membrane proteins that transport solutes, such as ions and metabolites maintain ionic homeostasis using ion or electrochemical gradients^15^. Over 300 human SLCs have been identified and studied with regards to their pharmacological applications as key proteins for drug transport^16^. Thus, research focusing the ion transporters in mosquito SGs will be useful not only for increasing our knowledge of these biological processes, but also for the development of new interventions for transmission blocking^17^.

In the present study, SGs were isolated from mosquitoes fed on *P. berghei*-infected and on non-infected mice, and RNA sequencing (RNA-seq) was performed. Gene expression levels were determined and compared between the infected and non-infected sialomes. The membrane ion transporters *prestinA* and *NDAE1* were found to be up-regulated in response to infection. Based on the gene expression during *Plasmodium* infection, putative co-interaction and transmembrane function to maintain cell homeostasis, these genes were selected to perform silencing experiments. Further gene silencing of *prestinA* and *NDAE1* resulted in significantly reduced vector survival and decreased sporozoite numbers in the SG. This work completes the analysis of the first Illumina-derived mRNAs assembly from *P. berghei*-infected *A. stephensi* SGs, constituting a valuable tool for future research^18^. Our present results suggest that membrane ion transporters in *A. stephensi* SGs affect *Plasmodium* infection, potentially representing new targets for development of measures to block malaria transmission.

## Results and Discussion

### Expression profiling and functional annotation of *A. stephensi* sialotranscriptome during *P. berghei* infection

Recently‚ it was demonstrated that, according to the system under study, transcriptomic changes are highly correlated with protein levels^19, 20^. Thus, the analysis of the transcriptomic response of *A. stephensi* SG to *P. berghei* infection can predict, at some extend, proteome regulation. Previous studies have investigated the *Anopheles* sp. sialotranscriptome^12, 21–24^, but concerning *A. stephensi*, there is only a previous work reporting an immune related response to *P. berghei* infection^18^. Herein, RNA-seq analysis enabled the production of two catalogs of transcripts corresponding to the *P. berghei* infected and non-infected SGs, which constitute a body of fundamental information to help with the selection of potential key targets for malaria control.

From the RNA sequencing data, we obtained a total of 26,357 and 36,393 transcripts for control and infected samples, respectively. Among these transcripts, 2536 showed differential expression in response to *P. berghei* infection (*P* < 0.05, Biological Coefficient Variation value = 0.2), of which 1996 (79%) were upregulated and 540 (21%) were downregulated. These findings suggest a vast network of interactions between the vector and parasite, with certain molecules likely being essential for parasite maintenance in the mosquito and/or for part of the mosquito’s response to infection. Other studies have shown comparable results^25, 26^.

**Figure 1:**
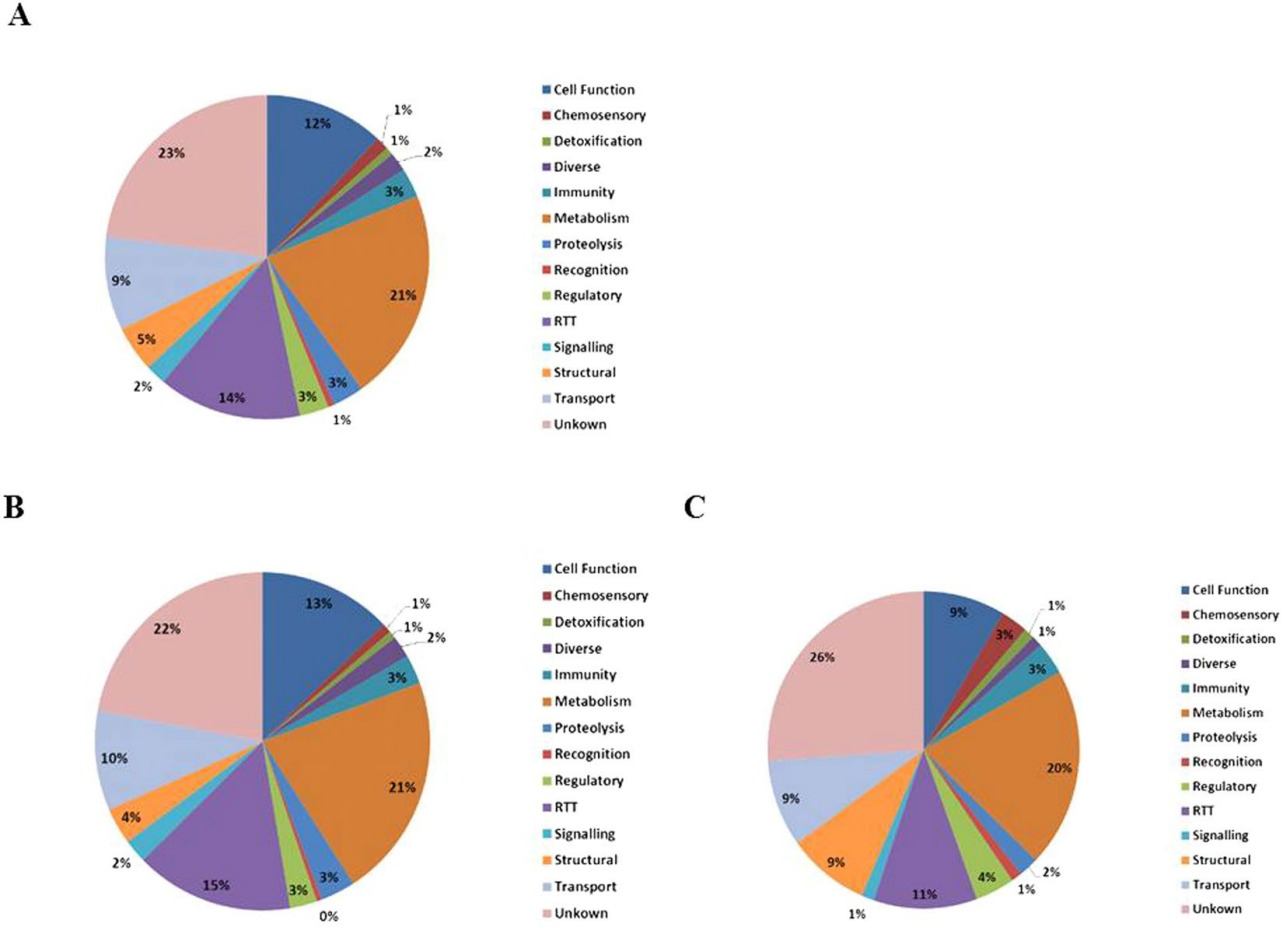
Differentially expressed genes in *Anopheles stephensi* salivary glands in response to *Plasmodium berghei* infection. (**A**) Distribution of differentially expressed transcripts by functional classes. (**B** and **C**) Distribution of upregulated (**B**) and downregulated (**C**) transcripts by functional classes. For annotation, gene identifiers were obtained from VectorBase (www.vectorbase.org) and compared to the corresponding sequences in *A. gambiae*.

The differentially expressed *A. stephensi* transcripts were functionally annotated using Gene Ontology (GO) terms, and were grouped into 14 classes: cell function, chemosensory, detoxification, diverse, immunity, metabolism, proteolysis, recognition, regulatory, replication-transcription-translation (RTT), signaling, structural, transport, and unknown function. Figure 1 presents the number of transcripts in each functional class, and their expression. Due to the importance of the innate immune response of anophelines to *Plasmodium* infection, the data concerning immunity class was discussed elsewhere^18^.

The assembly of differentially expressed *A. stephensi* SG transcripts revealed that the more commonly represented functional classes in this data set included metabolism, RTT, cell function, and transport (539, 363, 303, and 238, respectively), while less common functional classes included recognition, detoxification, and chemosensory (16, 20, and 33, respectively) (Fig. 1A). The most over-expressed and under-expressed transcripts were found in metabolism, RTT, cell function, and transport. Among the metabolism class transcripts, 429 were upregulated while 110 transcripts were downregulated. Of the transcripts of the RTT and cell function classes, respectively, 305 and 257 transcripts were upregulated while 58 and 46 were downregulated. Regarding the 238 transport class transcripts, 190 were upregulated and 48 downregulated. As SLCs play a role in *Plasmodium*–SGs interactions hence here main preference was given to this class that includes 33 upregulated transcripts and 5 downregulated transcripts.

### Validation of RNA-seq results

For validation of the RNA-seq data, qPCR was conducted. Fourteen genes that were differentially expressed in response to *Plasmodium* infection, including 8 that were upregulated (fold-change of 5.67 to 1.49) and 6 that were downregulated (fold-change of −3.16 to −1.39), were analyzed (see Supplementary Table S1). There was a strong correlation between the mRNA levels obtained by both RNA-seq and qPCR methods (Pearson’s correlation coefficient = 0.667, *P* = 0.009) (see Supplementary Fig. S1).

#### Metabolism genes

Changes in metabolism-related transcripts may be associated with increases of metabolites favoring sporozoite development within SGs before transmission to a vertebrate host^22, 27^. Such alterations could also be related to SG maintenance and repair after *Plasmodium* invasion. Metabolism was the most enriched functional class, accounting for 21.49% of upregulated genes and 20.37% of downregulated genes. Metabolic processes can be induced by parasites, for example, *de novo* biosynthesis of pyrimidines is required by *P. falciparum* ^28^. Our present data included a fold-change of 4.17 in the transcript AGAP004172, which is described as showing orotate phosphoribosyltransferase (OPRT) activity mediating this metabolic pathway^28^. Another transcript, AGAP003184 (fold-change of 2.67), reportedly plays a role in the heme-biosynthetic pathway favoring *Plasmodium* survival^29, 30^.

Previous studies show that chondroitin glycosaminoglycan presence in the apical midgut microvilli of *A*. *gambiae* enables parasite adhesion^31, 32^, suggesting its involvement in the metabolic process. Herein, the chondroitin 4-sulfotransferase represented by AGAP005721 was upregulated in *P. berghei*-infected SGs, supporting the possibility that this molecule may be related to parasite adhesion to SGs.

#### RTT genes

Translational control of gene expression may result from cell stress and could allow cells to regulate protein levels in relation to infection processes. A total of 363 transcripts from the RTT functional class were differentially expressed, including 15.28% of upregulated and 10.74% of downregulated transcripts. Increased translational regulation has also been observed in *P. falciparum-*infected *A. gambiae* ^33^ and in the SGs of *P. berghei*-infected *A. coluzzii* ^5^. Moreover, differential transcription has been detected in hemocytes of *P. berghei-*infected *A. coluzzii* ^34^, and a high number of upregulated genes related to DNA replication were found in dengue virus-infected *A. aegypti* ^35^. It is possible that the *Plasmodium* parasite may interfere with or manipulate the expressions of genes required for invasion and development within the SGs.

#### Immunity genes

The results concerning the immune response were discussed in a previous study conducted by the authors^18^. Since the innate immune response is activated when pathogens are recognized, it is expected that this class will be strongly represented in studies focusing on infection.

#### Transport genes

Membrane transport proteins mediate the passage of specific molecules and/or ions across the membrane bilayer of the SGs, controlling nutrient uptake, removal of metabolic wastes and xenobiotics, and the maintenance of transmembrane electrochemical gradients^36^. This activity also ensures that SGs meet the requirements for *Plasmodium* survival^15, 37^.

A SAGE analysis revealed that *P. berghei* infection impacts the expression of transport-related transcripts, suggesting that the parasite may exploit *A. gambiae* cellular mechanisms. Moreover, a microarray-based experiment revealed the upregulation of 326 transport-related genes among a total of 4978 transcripts from *A*. *gambiae* SGs after *P. berghei* infection^23^.

Our present results showed that transport-related transcripts were among the most differentially expressed (238 transcripts of the total of 2536), with 9.52% upregulated and 8.89% downregulated. This is in accordance with a previous report in *A. coluzzii* ^5^. Within this category, 50 transcripts were identified as ion transmembrane transporters. These proteins mediate the passage of specific molecules and/or ions through SGs membrane, and are associated with a diverse range of physiological roles, including the maintenance of transmembrane electrochemical gradients^36^. In particular, we identified transcripts related to the solute carrier family (SLC), including AGAP007054 (SLC13), AGAP007743 (SLC16), AGAP005405 (SLC39), AGAP000097 (SLC25), AGAP005537 (SLC35), AGAP010725 (SLC26), and NDAE1 (SLC4). Based on the influence and shared function of transporters in mosquito SGs during infection, and the high expression levels of such genes in our data, we selected the solute carriers SLC26 and NDAE1 as targets for further studies. These molecules are described below in greater detail.

#### Other functional classes

Some genes that were identified in this study belonged to functional classes other than those mentioned above, and have been previously reported as associated with *Plasmodium* infection. The transcript AGAP010035 from the cell function class was upregulated (fold-change of 2.34), and is related to the TOR nutritional hormone signaling pathway, which is affected by parasite infection in the midgut^38^. Additionally, we detected downregulation of two salivary gland surface (SGS) proteins, SGS4 (fold-change of −1.51) and SGS5 (fold-change of and −2.75), which are considered highly immunogenic and related to both blood feeding and parasite infection^39, 40^, suggesting parasite expression regulation.

Proteins from the proteolysis class are involved in several proteolytic events during blood feeding, including digestion of blood, sugar, and extracellular matrix components^23, 41^. The proteolysis gene *AGAP009212* (fold-change of 1.49)—known as SRPN6, a malaria parasite invasion marker in mosquitoes—suggests that serpins are associated with the immune response against *Plasmodium* infection in *A. stephensi*. In fact, mosquito serpins control melanization^42–45^ and host homeostasis during blood feeding^44^. Previous reports showed that serine proteases are also related to dengue virus transmission in *Aedes* mosquitoes^46^.

Apoptosis, i.e., programmed cell death, is a crucial cellular mechanism for tissue homeostasis and normal immune system functioning, which is highly regulated by a variety of caspases, Bcl-2 proteins, and inhibitors of apoptosis (IAPs)^47^. Studies using *P. berghei* reveal the induction of cell death and apoptosis during infection^48, 49^. Our present data showed upregulation of the initiator caspase CASPL2 (fold-change of 0.95), which is reportedly related to apoptosis during parasite infection. Ramphul *et al*.and collaborators (2015) silenced initiator caspases, such as CASP-L1, CASP-L2 and IAP1, and reported no significant changes in *Plasmodium* infection intensity or infection prevalence among mosquitoes^50^. Downstream substrates of caspases, such as Bcl proteins, have been found in the *A. stephensi* sialome. Here we detected upregulation of Anob-1 (AGAP011552) (fold-change of 1.44), which is a protein from the Bcl-2 family (DEBCL) that acts as an apoptosis regulator and is related to autophagy^51^.

#### PrestinA and NDAE1 interactions

The *prestinA* and *NDAE1* genes, respectively, were annotated as encoding a protein with sulfate transmembrane transporter activity (Gene Ontology annotation: http://www.ebi.ac.uk/QuickGO/GTerm?id = GO:0015116) and a protein with anion transmembrane transporter activity (Gene Ontology annotation: http://www.ebi.ac.uk/QuickGO/GTerm?id = GO:0008509). PrestinA is an anion exchanger belonging to the SLC26 family, while NDAE1 participates in anion transport and belongs to SLC4 family^15^. The transmembrane localizations of prestinA and NDAE1 were confirmed using location predictor software.

**Figure 2:**
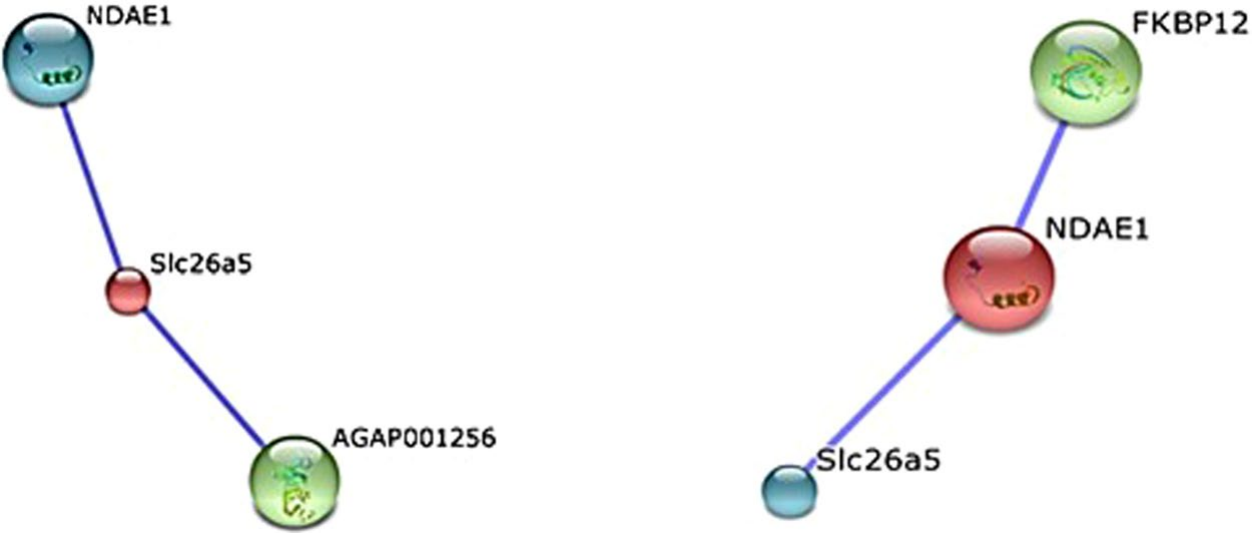
Predicted networks of selected solute carriers. STRING-based network prediction analysis of the *prestinA* and *NDAE1* genes, respectively, considering a minimum required interaction score of 0.700 (high confidence).

Prestin transporters have been identified in *Drosophila melanogaster* (*dPrestin)* and *A. gambiae* (*AgPrestinA* and *AgPrestinB*) as homologues of mammalian *SLC26a5*. *AgPrestinA* and *AgPrestinB* are highly expressed in the SGs and midgut, respectively^15, 52^. In our data, the mosquito orthologue was found over-expressed (RNA-seq: fold-change of 2.19; qPCR: fold-change of 4.70) in *Plasmodium*-infected SGs. PrestinB is a transmembrane protein that supports the exchange of ions HCO^3–^, oxalate^2−^, SO4^2−^, and formate across the midgut membrane^15^. Uptake of SO_4_^2−^ by prestinB is essential for biosynthesis of chondroitin sulfate (ChSO_4_), an epithelial ligand that allows midgut invasion^31^. PrestinB also mediates the uptake of bicarbonate produced by carbonic anhydrase or supplied by Na^+^-driven anion exchanger (NDAE1)^53^ that was also found to be over-expressed (RNA-seq: fold-change of 3.77; qPCR: fold-change of 1.98) as revealed in our studies. NDAE1 is a solute carrier that plays a central role in bicarbonate, sulfate, and oxalate metabolism, contributing to maintaining pH homeostasis in the cell lumen. Disruption of this gene is reportedly lethal to *Drosophila* larva^54^. As expected, both these SLC transcripts were found over represented in the previously reported sialotranscriptome of *A. coluzzii* ^5^. Moreover, both SLC are presented in both *A. gambiae* (NCBI (https://www.ncbi.nlm.nih.gov/) with the Accession no. UP_000007062) and *A. stephensi* (VectorBase (https://www.vectorbase.org/) with the Accession no. AsteI2.3.) proteomes, which indicates the possibility of these transporters being expressed in the mosquito. We further analyzed the potential interaction between these two solute carriers using STRING software for prediction of protein–protein interaction (Fig. 2).

*PrestinA* and *NDAE1* showed close homology to the *A. gambiae* genes AGAP010725 and AGAP009736, respectively, with higher bitscores and lower E values. PrestinA (SLC26a5; 641 amino acids) may interact with at least two possible network partners: the proteins NDAE1 and K13811. NDAE1 (AGAP009736) showed the closest relationship, with a high confidence score of 0.729. The protein K13811, a 3′-phosphoadenosine 5′-phosphosulfate synthase (666 aa) encoded by the AGAP001256 gene, showed an interaction score of 0.731 with prestinA. In sulfation reactions, K13811 is considered an obligate cosubstrate for active sulfate^55^. Remarkably, our data showed that the AGAP001256 transcript was also upregulated (fold-change of 1.86). NDAE1 network predictions indicated the existence of two proteins: prestinA (confidence score of 0.729) and FKBP12 (108 aa; confidence score of 0.753), which was not present in the transcriptomic data.

**Figure 3:**
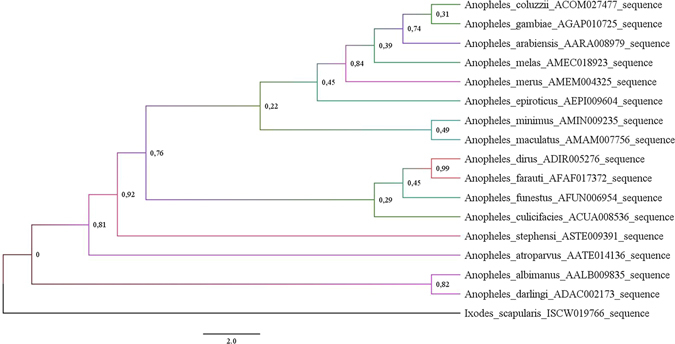
Prestin molecular phylogenetic analysis by Maximum Likelihood method. Using the Maximum Likelihood method based on the best fitted models (LG + G), the tree with the highest log likelihood (−1660.211) is shown. A Gamma distribution (G) was used to model evolutionary rate differences among sites (5 categories ( + G, parameter = 0.79). The tree is drawn to scale, with branch lengths measured in the number of substitutions per site. The analysis involved 17 amino acid sequences with respective VectorBase accession number: *A. albimanus* (AALB009835); *A. arabiensis* (AARA008979); *A. atroparvus* (AATE014136); *A. coluzzii* (ACOM027477); *A. culicifacies* (ACUA008536); *A. darlingi* (ADAC002173); *A. dirus* (ADIR005276); *A. epiroticus* (AEPI009604); *A. farauti* (AFAF017372); *A. funestus* (AFUN006954); *A. gambiae* (AGAP010725); *A. maculatus* (AMAM007756); *A. melas* (AMEC018923); *A. merus* (AMEM004325); *A. minimus* (AMIN009235); *A. stephensi* (ASTE009391) and *Ixodes scapularis* (ISCW019766) (outgroup). All positions containing gaps and missing data were eliminated and bootstrap values for internal branches are shown.

Furthermore, the evolutionary history of the prestin (Fig. 3) indicates a high probability that the *A. stephensi* protein is closely related to the majority known proteins from other malaria vectors.

**Figure 4:**
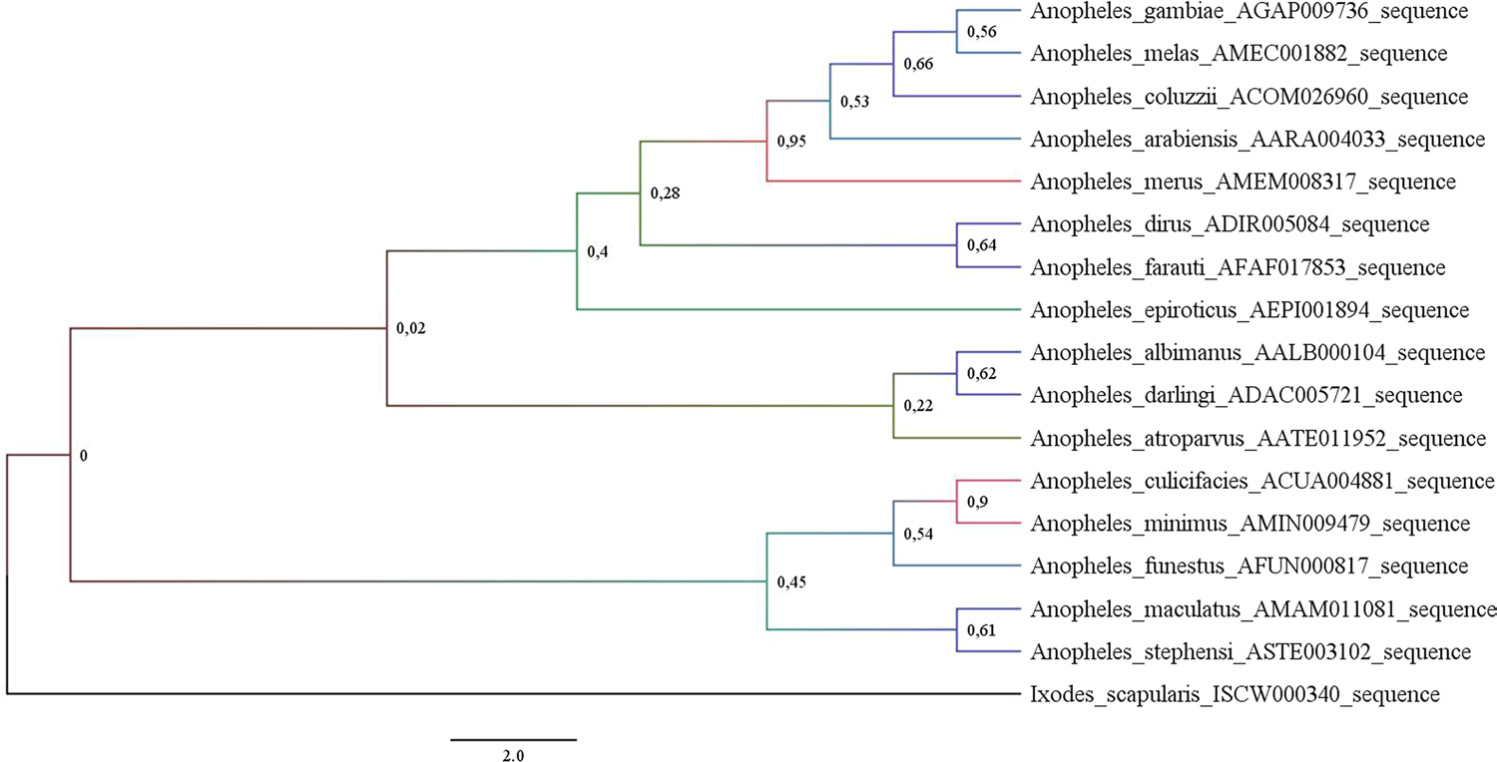
NDAE1 molecular phylogenetic analysis by Maximum Likelihood method. Using the Maximum Likelihood method based on the best fitted models (LG + G), the tree with the highest log likelihood (−4016.814) is shown. A Gamma distribution (G) was used to model evolutionary rate differences among sites (5 categories ( + G, parameter = 0.27). The tree is drawn to scale, with branch lengths measured in the number of substitutions per site. The analysis involved 17 amino acid sequences with respective NCBI accession number: *A. albimanus* (AALB000104); *A. arabiensis* (AARA004033); *A. atroparvus* (AATE011952); *A. coluzzii* (ACOM026960); *A. culicifacies* (ACUA004881); *A. darlingi* (ADAC005721); *A. dirus* (ADIR005084); *A. epiroticus* (AEPI001894); *A. farauti* (AFAF017853); *A. funestus* (AFUN000817); *A. gambiae* (AGAP009736); *A. maculatus* (AMAM011081); *A. melas* (AMEC001882); *A. merus* (AMEM008317); *A. minimus* (AMIN009479); *A. stephensi* (ASTE003102) and *Ixodes scapularis* (ISCW000340) (outgroup). All positions containing gaps and missing data were eliminated and bootstrap values for internal branches are shown.

The molecular phylogenetic analysis showed that *A. stephensi* and *A. maculatus* NDAE1 proteins are in the same monophyletic group, being closely related (Fig. 4).

This high degree of conservation of prestin between anophelines emphasized the potential of this target to block malaria transmission in different malaria vector species.

Considering this information, we further used RNAi to assess the co-participation of these two SLCs, which share the same physiological processes and functions, in parasite invasion and transmission^52, 54^ in mosquito SGs.

### Knockdown of *prestinA* and *NDAE1*: Solute carrier pathways as a potential target for blocking malaria transmission

Membrane transporter proteins of mosquito SGs are considered potential candidates for the development of malaria control measures. For example, saglin is essential for *Plasmodium* sporozoites to invade the SGs^56^ and AGAP007752 is up-regulated in the *A. gambiae* sialotranscriptome after *P. berghei* invasion^5^. As mentioned above, we conducted further studies focusing on two SLCs based on their important roles as transmembrane transporters and co-interaction with *Plasmodium* and mosquito-cell survival. RNA interference-mediated gene silencing was used to evaluate the potential effects of *prestinA* and *NDAE1* on *A. stephensi* mosquitoes upon infection with *P. berghei*.

**Figure 5:**
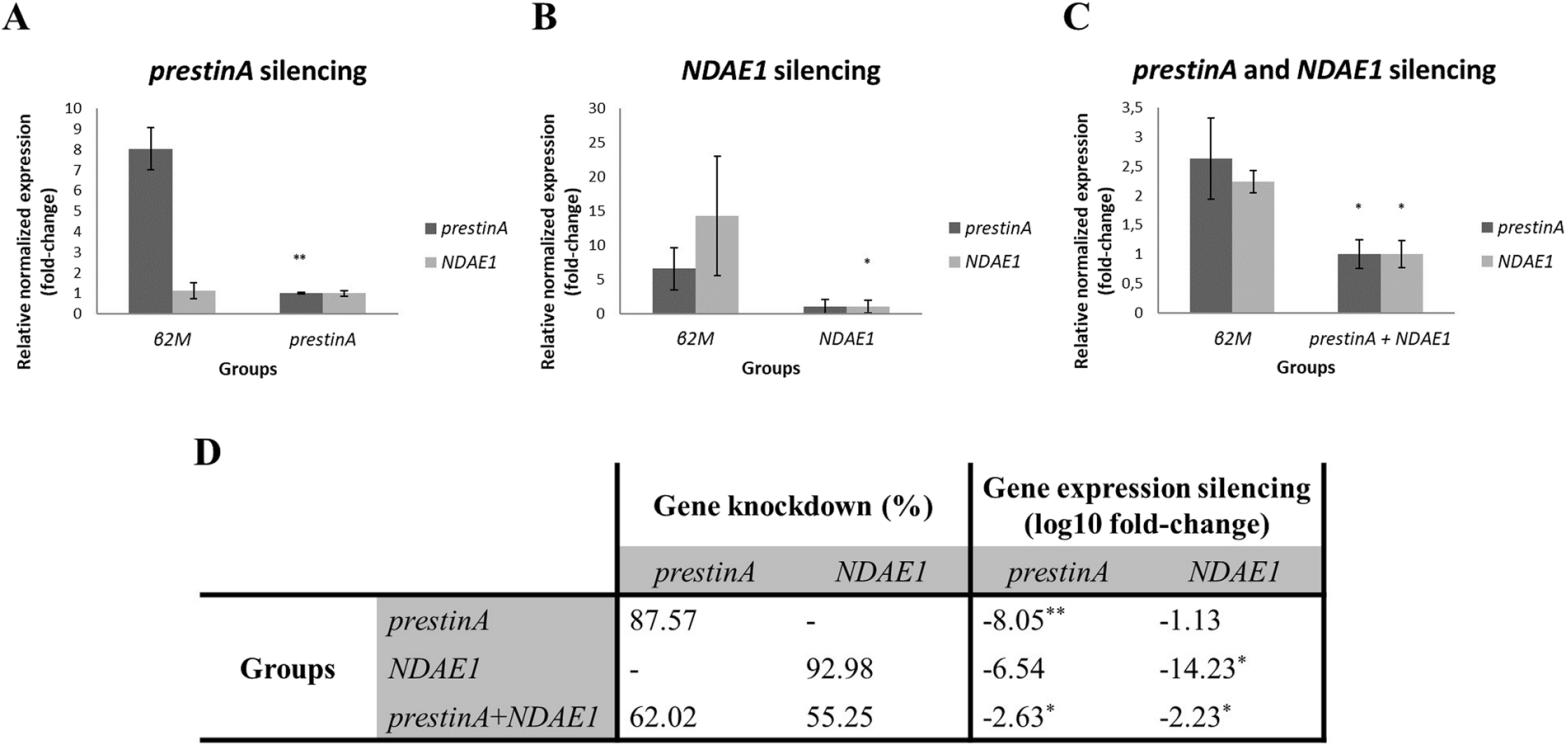
Results of *prestinA* and *NDAE1* gene knockdown in *Anopheles stephensi* salivary glands. (**A**–**C**) The relative expressions of *prestinA* and *NDAE1* were evaluated in *Anopheles stephensi* salivary glands with gene knockdown of *prestinA*, *NDAE1*, and in co-silenced groups, respectively. Normalized against *RPS7*, Cq values were compared between mosquitoes injected with dsRNA for the selected genes and with β2M control dsRNA. The expression of the genes in the *prestinA*, *NDAE1* and both silenced groups is set to 1 for a better interpretation. Statistical analyses were conducted using the Pfaff method. *P < 0.05, **P < 0.01, ***P < 0.001. (**D**) Summary of silencing assay results. Gene expression levels and gene knockdown percentage of the treated groups.

**Figure 6:**
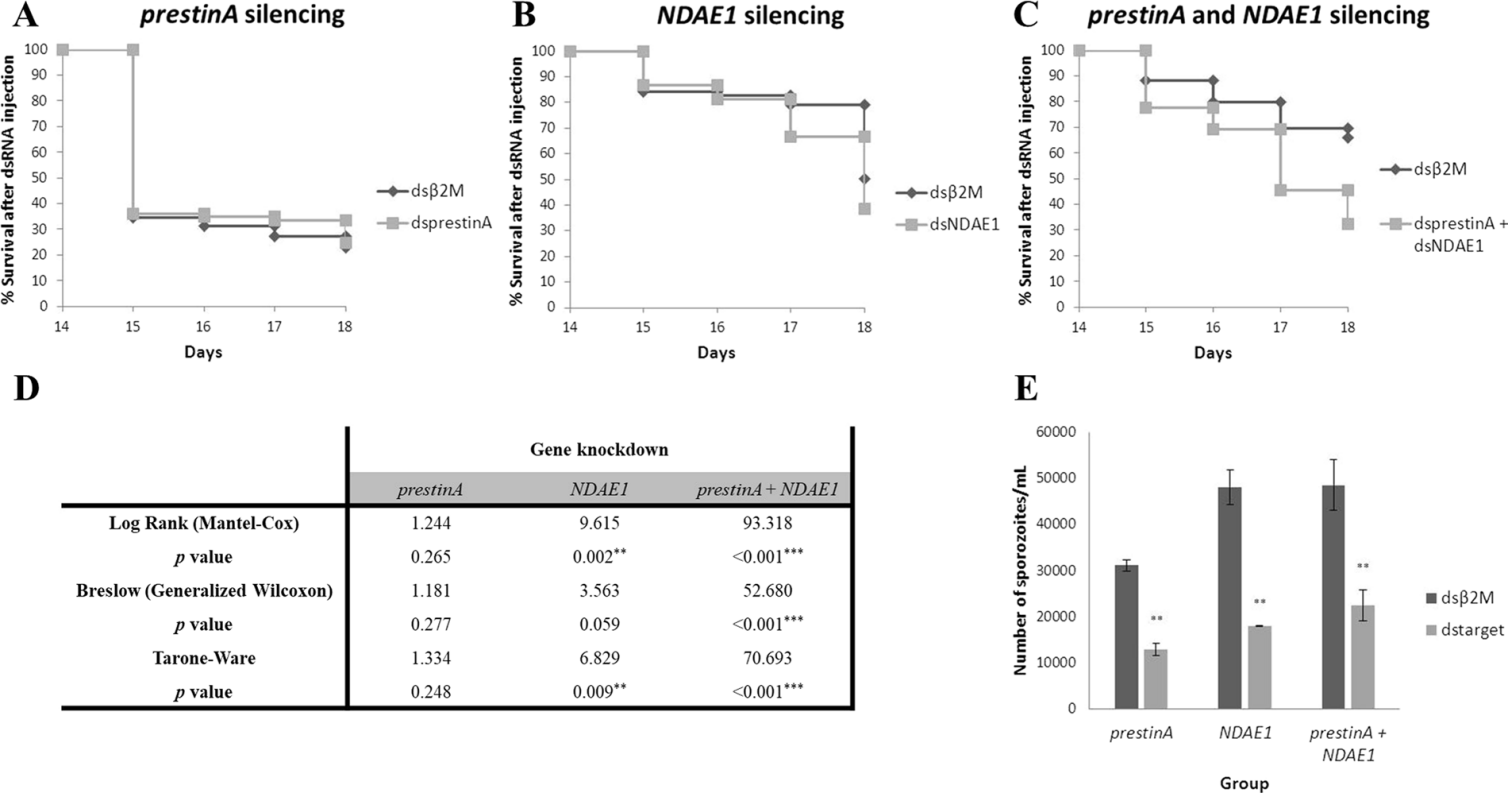
Knockdown of the *prestinA* and *NDAE1* genes in *Anopheles stephensi* salivary glands. (**A**–**C**) Kaplan-Meier plots of the survival of *Anopheles stephensi* mosquitoes after silencing of *prestinA* (**A**), *NDAE1* (**B**), or both genes together (**C**). (**D**) Kaplan-Meier statistics with values from the Log-Rank, Breslow, and Tarone-Ware tests. *P < 0.05, **P < 0.01, ***P < 0.001. (**E**) Effects of silencing *prestinA*, *NDAE1*, or both genes together in the *Anopheles stephensi* salivary glands with regards to the number of sporozoites. Distributions were compared using the Mann-Whitney test. *P < 0.05, **P < 0.01, ***P < 0.001.

For dsRNA synthesis, both fragments of interest were first amplified and further sequenced using the T7 promoter. The obtained sequences were compared to available databases using the Basic Local Alignment Search Tool (BLAST) from VectorBase. The products showed high similarity to the *prestinA* sequence (99.8% reverse primer and 96.3% forward primer) and the *NDAE1* sequence (99% reverse and forward primer). Synthetized dsRNA was checked for integrity, and ~276 ng of dsRNA was injected into each mosquito. The dsRNA was injected fourteen days post-infection because at this time point the ruptured oocyts release sporozoites which can then migrate to the salivary glands. After silencing assays, we evaluated the gene expression levels (Fig. 5A–C), percentage of gene silencing (Fig. 5D), survival rate (Fig. 6A–D), and numbers of sporozoites on SGs (Fig. 6E).

In Fig. 5, the relative expression of *prestinA* and *NDAE1* genes is represented in the *prestinA* (Fig. 5A), *NDAE1* (Fig. 5B), and co-silenced *A. stephensi* groups (Fig. 5C). Mosquitos injected with *prestinA* dsRNA showed 87.57% gene knockdown, significantly reducing the *prestinA* expression level (−8.05; *P* = 0.005). *NDAE1* was knocked down by 92.98%, significantly reducing the gene expression level (−14.23; *P* = 0.047). *PrestinA* and *NDAE1* expression levels were also evaluated under the effect of knockdown of the other gene. *PrestinA* silencing was associated with a decline of *NDAE1* mRNA levels (−1.13; *P* = 0.264), and *NDAE1* silencing was associated with reduced *prestinA* expression (−6.54; *P* = 0.271), suggesting interaction between these two proteins. Double knockdown (silencing 62.02% of *prestinA* and 55.25% of *NDAE1*) resulted reduced mRNA expression levels for both genes (*prestinA* = −2.63, *P* = 0.018; *NDAE1* = −2.23, *P* = 0.012) compared with the control group. The lower silencing efficiency in this case can be explained by the amount of specific dsRNA used, since in the double knockdown, we inoculated mosquitoes with ~138 ng/injection of each dsRNA.

For each group, the survival rate was recorded daily until the 18th day PBM (Fig. 6A–C), and compared to the *β2M*-dsRNA-injected controls.

Under the conditions of the present study, our results showed that *prestinA* silencing seems to not influence the survival of *A. stephensi* mosquitoes (P > 0.05) (Fig. 6A,D). Since an abnormal mortality in the initial time points, in both control and *prestinA* silenced groups was observed data was re-tested excluding the 14^th^ and 15^th^ day. Nevertheless, statistical analysis continues to point to a weak effect of *prestinA* silencing in mosquito survival (see Supplementary Table S4). This suggests that other molecules can compensate for the cellular absence of *prestinA*. Other studies of different transporters, such as apolipophorin-III (a lipid transporter) and fondue (a component of the hemolymph clot), show that manipulation of their gene expressions in *A. gambiae* G3 strain mosquitoes resulted in survival patterns similar to that of the control^57^. However fondue-silenced mosquitoes reportedly influence fungal infections^57^. Our results showed that double knockdown profoundly affected mosquito mortality, with a 23% population reduction. Moreover, knockdown of *NDAE1* alone led to a 51% population reduction (*P* < 0.05) (Fig. 6B–D). This influence of NDAE1 was also detected in a study of *Drosophila* larvae, in which NDAE1 was found to be crucial for fly development^54^. Our present results support this molecule’s vital role in Cl^−^/HCO_3_^−^ exchange as a component of the steady-state cell alkalization in the mosquito^58^. Based on previous findings that acute intracellular pH acidification can rapidly kill a cell, targeting cellular acidification is being considered as a method of cancer therapy^59^. Recently, a study showed that the survival rate of transgenic mosquitos lacking SGs was not affected^8^, which could point to a weak effect of *NDAE1* knockdown in mosquito mortality, but NDAE1 is not exclusive to SGs^15^ and therefore, manipulating its expression in the whole organism had a visible physiological effect (lower survival rate). On the other hand, *prestinA* is SG exclusive, and thus the disruption of mRNA is tissue specific.

To evaluate infection levels, sporozoites were quantified at four days post-dsRNA injection. All knockdown groups showed statistically significant reduction of sporozoite numbers. Knockdown of *prestinA* reduced infection by 42.2% (*P* = 0.003), the *NDAE1* silenced group showed an infection reduction of 73.5%, (*P* = 0.006), and double knockdown resulted in a reduction of 53.2% (*P* = 0.001) (Fig. 6E).

These results together with previous findings^15, 31^, support that reducing transporter expression in *A. stephensi* SGs, and thus lowering sulfate uptake and leading to deficient chondroitin sulfate biosynthesis, could prevent *Plasmodium* invasion by reducing the ability for parasite adhesion.

**Figure 7:**
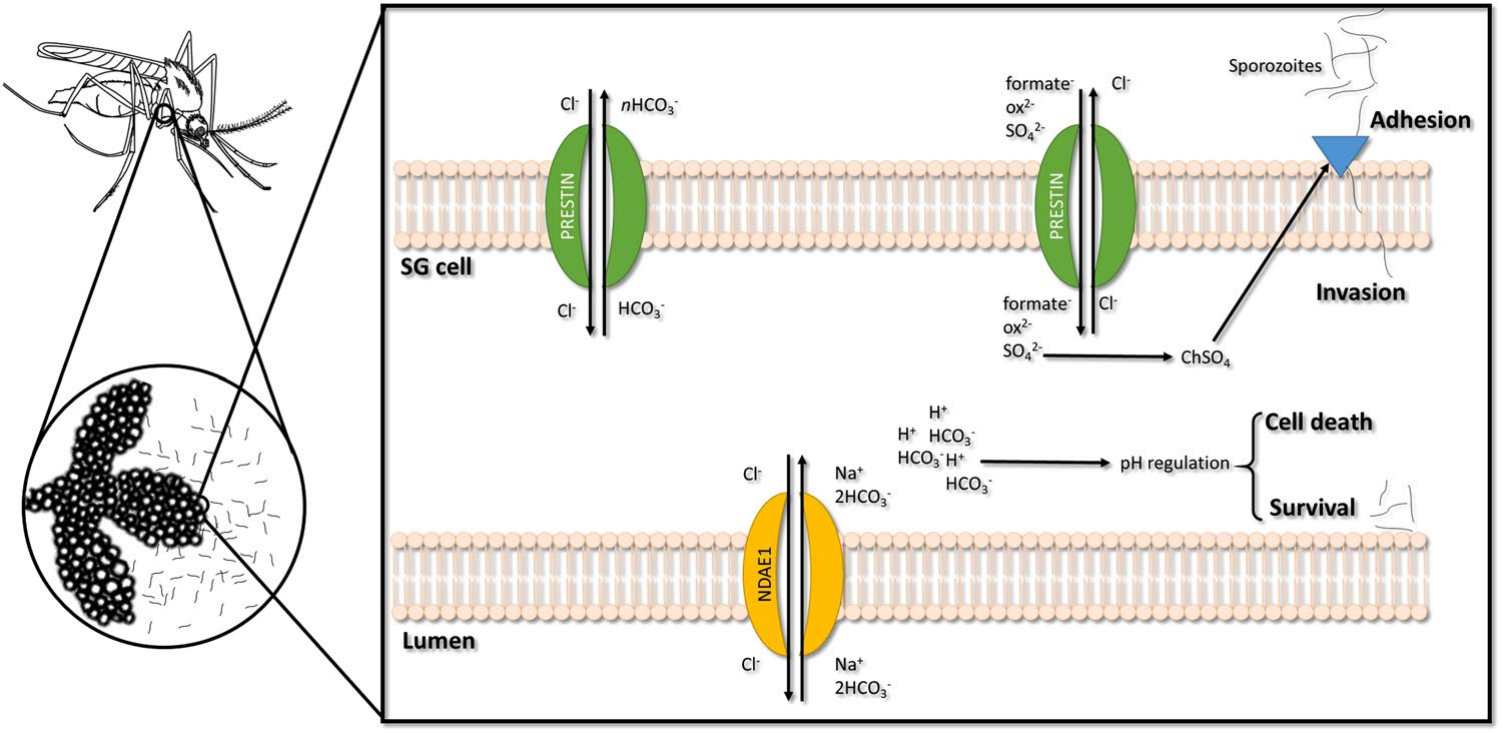
Proposed model for prestinA and NDAE1 physiological functions and impact on SG cell and *Plasmodium berghei* infection. PrestinA is expressed at apical membrane of cells, mediates the uptake of sulfate and excretes bicarbonate which is produced by carbonic anhydrase or supplied by NDAE1. Disruption of prestinA alters the biosynthesis of chondroitin sulfate which interferes with *Plasmodium* adhesion. A reduction of both prestinA and NDAE1 changes cell pH, affecting *Plasmodium* and mosquito survival.

Sustained pH homeostasis is essential for viable *Plasmodium* invasion/multiplication in mosquito tissues^60^. Manipulation of *NDAE1* expression in the whole vector may produce a synergetic effect, resulting in an acute reduction of sporozoites in SGs due to pH alteration. Silencing of both genes may have altered system equilibrium, exposing *P. berghei* to different/adverse environmental parameters. Figure 7 presents our hypothesis of prestinA and NDAE1 physiological function in *A. stephensi*-SGs cells and their influence on *Plasmodium* infection.

Suppression of ion and solute exchangers can disrupt different metabolic pathways; therefore, these transmembrane proteins may be useful in new transmission-blocking approaches. The impact of trehalose and glucose transporters has already been demonstrated in terms of survival of *A. gambiae* ^37^ and *A. coluzzii*, as well as *P. berghei* maintenance in SGs. Our present findings provide crucial information for the selection of target genes for preventing malaria transmission, including our report of the *A. stephensi* sialotranscriptome during *P. berghei* infection and our functional characterization of *prestinA* and *NDAE1*. These ion and solute membrane transporters are tightly connected, and were demonstrated to be upregulated after infection. Moreover, RNAi-mediated gene knockdown results suggested that the proteins encoded by these genes may affect *Plasmodium* viability in the SGs. Further investigation is needed to improve our understanding of basic transport, transport regulation, and how these processes regulate vector–parasite interaction. To eradicate and control malaria disease, it is crucial that we explore new targets for developing control measures and transmission-blocking vaccines.

## Methods

### Ethics statement

Animals were maintained at the Institute of Hygiene and Tropical Medicine (IHMT), in accordance to Europe Directive 86/609/ EEC and Portuguese law (Decreto-Lei No. 129/92). Animal experiments were conducted with the approval of the Divisão Geral de Alimentação e Veterinária (DGAV), Portugal, under Art° 8, Portaria n°1005/92 from 23^rd^ October (permit number n° 023357) and the Council of Ethics of the IHMT. All experiments were performed in accordance with relevant guidelines and regulations.

### Mosquito rearing

The *A. stephensi* mosquitoes were obtained from the IHMT insectary and reared at 20 °C in 70% humidity, under a 12 h light/dark photoperiod. Adult mosquitoes were fed *ad libitum* on a 10% glucose solution. For experiments, only mosquitoes aged between 3–5 days were used.

### *Plasmodium* infection

To validate gene expression and gene knockdown efficiency, mosquitoes fed on uninfected and *P. berghei* -infected mice were obtained. Female CD1 mice were intraperitoneally inoculated with 10^7^ *P. berghei* ANKA parasitized red blood cells. The levels of parasitaemia were measured from blood smears of the mouse tail using light microscopy after staining with Hemacolor^®^ kit (EMD Millipore, Germany). Mice were used when the parasitaemia reached about 10% and exflagellation was observed (4–6 exflagellations/field).

Three hundred female mosquitoes per group were allowed to feed on anesthetized mice, and only fully engorged females were kept at 19–21 °C and 80% humidity.

### RNA extraction

Midguts and SGs were dissected from cold anaesthetized female mosquitoes, under a stereoscopic microscope at 4X magnification (Motic SMZ-171B, China). Detailed methodology of tissue and RNA isolation is described elsewhere^5^. In order to check *Plasmodium* infection, midguts were dissected at day 8–9 post-blood-meal (PBM) confirming the presence of oocysts; at day 18 PBM, SGs were extracted and kept either in ice cold PBS for sporozoites quantification or in ice-cold RNA later (Ambion, Austin, TX, USA) for RNA extraction.

RNA was promptly extracted using RiboZol™ (AMRESCO, USA) and stored at −80 °C. The quantification and integrity of the RNA was assessed using a ND-1000 Spectrophotometer (NanoDrop ND1000, Thermo Fisher Scientific, Whaltman, MA) and a Agilent 2100 Bioanalyser® system (Agilent Technologies, USA), respectively.

### RNA sequencing

RNA-seq was conducted in SGs of mosquitoes fed on uninfected and *P. berghei*-infected mice. Individual SGs of from each condition were grouped to obtain the quantity needed to perform high throughput sequencing. Sequencing was performed at Parque Científico de Madrid (FPCM, Spain). From 1 µg of total-RNA, enrich poly (A) mRNA were used to bead with magnetic beads with Oligo (dT) to construct the cDNA library. Next, from purified mRNAs, two different fragmentation conditions were applied so that a “shorter” and a “longer” preparation was made for both control and infected RNA samples. Further, double-stranded cDNAs were synthesized and subjected to end-repair, adenylation, ligation with sequencing adapters and PCR amplification. Proper fragments were purified by size using AMPure XP beads (Beckman Coulter, High Wycombe, UK) and size and quality confirmed using an Agilent High Sensitivity DNA kit (Agilent Technologies, CA, USA). The size range of fragments was around 310 bp for the short insert and 465 bp for the longer insert preparations. Next, the libraries were pooled and titrated using qPCR to get an accurate estimation of concentration. In a Cluster Station (Illumina, CA, USA), clusters were generated and using a GAIIx equipment (Illumina, CA, USA), the libraries were sequenced using a 2 × 100 cycle sequencing run program separated by a paired-end turnaround. TruSeq RNA kit (Illumina, CA, USA) was used to prepare cDNA library as per manufacturer instructions. Based on the results provided by FPCM, Era7 Company performed the transcriptomic analysis. Trimmed sequences were used for de novo assembly using Oases program and functional information obtained from Uniprot (GO annotations, Enzyme Commission (EC) number, Interpro motifs). For each condition, Bowtie and Samtools were used to map and determine the RPKM values of reads. After clustering transcripts, eXpress program was used to obtain the quantification per protein, and each set of transcripts annotated by the same protein was considered as a Unigene provided with functional annotation. Differential expression analysis of Unigenes in infected versus uninfected mosquitoes was carried out using EdgeR^61, 62^. For this data, each transcript compiles information of UniProt, RefSeq, GO, VectorBase and NCBI Taxonomy Enzyme databases. After the initial analysis, transcripts were functionally annotated into 14 classes: cell function, chemosensory, detoxification, diverse, immunity, metabolism, proteolysis, recognition, regulatory, RTT, signaling, structural, transport and unknown. Previous studies have investigated the sialotranscriptome *of A. stephensi* regarding exclusively the immunity class^18^. The remaining data is analyzed in the present study.

Up or down gene regulation was also considered in this analysis. RNA-seq data can be found in the database: ArrayExpress (www.ebi.ac.uk/arrayexpress) with the Accession no. E-MTAB-3964.

### Validation of transcriptomics data

Fourteen transcripts identified by RNA-seq of *A. stephensi* SGs with differential regulation and belonging to different functional classes with a potential interference in the parasite development, were chosen for RNA-seq validation through relative qPCR. Using three biological replicates of each condition (infected and non-infected), total RNA (100 ng/µL of each sample) were used to synthesize cDNA using the iScript™ cDNA Synthesis Kit (Bio-Rad, CA, USA). qPCR reactions of 20 μl were performed in triplicate using IQ^™^ SYBR^®^ Green Supermix kit (Biorad, CA,USA) in a CFX96 Touch Real-time PCR (Bio-Rad, CA, USA) thermocycler. The following conditions were used: an initial cycle of denaturation at 95 °C for 10 min; followed by 45 cycles of 95°C for 15 seconds and temperature of each primer set for 45 seconds. Fluorescence readings were taken at 62 °C after each cycle and a melting curve (60–95 °C) was performed. The sequences of the primers were design using Primer3 platform (http://bioinfo.ut.ee/primer3–0.4.0**/**) and their conditions are listed in Supplementary Table S2.

Standard curves were constructed with 10-fold serial dilutions of cDNA synthetized from total RNA from a pool of eight females kept exclusively on a sugar diet, in order to determine the reaction efficiency. To ensure that only one amplicon was formed and the amplicons denatured at the same temperature, a dissociation curve was run at the end of the reactions. Relative expression levels were normalized using the *ribosomal protein S7* (*RPS7*) (Vectorbase: ASTE004816) as reference gene (see Supplementary Table S2) and the *Pfaff* method for analysis^63^. Normalized Cq values were compared between infected and control samples by Mann-Whitney test (P < 0.05). Pearson’s correlation was used to compare the expression values between RNA-seq and qPCR methods for the 14 selected genes (see Supplementary Fig. S1).

### Network and localization analysis

Genes selected for functional analysis were further characterized using the software STRING 10.0 (Search Tool for the Retrieval of Interacting Genes/Proteins available at http://string-db.org) in order to identify known/novel protein-protein interactions. Since STRING software until date only accepts *A. gambiae* data, orthologues from *A. stephensi* obtained sequences were used^7, 64^. Briefly, the program by single protein name or amino acid sequence generates the network images based on a spring model. To predict transmembrane domains/protein localization, TMHMM, based on a hidden Markov model^65, 66^, WoLF PSORT^67^ and MultiLoc2^68^ were applied to amino acid sequences.

### Sequence analysis

From VectorBase database (https://www.vectorbase.org/), the amino acid sequence of the selected SLC proteins were used to search for the orthologues (https://www.vectorbase.org/blast). The sequences of the orthologues and main vectors of malaria were obtained. *Ixodes scapularis*-related sequence was used as outgroup. All sequences were aligned with MAFFT (v7) (Katoh & Standley 2016) and all positions containing gaps were exclude with GUIDANCE2 (Sela *et al*. 2015). Molecular Evolutionary Genetics Analysis (MEGA, version 6) software was used to obtain the best model to build^69^ a Maximum Likelihood tree based on a bootstrapping method with 1000 replicates. Generated trees were visualized and edited using FigTree v1.4.3^70^.

### RNAi

RNAi-mediated gene-silencing assays were performed to evaluate the effect of the knockdown of the genes *prestinA* (ASTE009391) and *NDAE1* (ASTE003102) on *P. berghei*-infected *A. stephensi* mosquitoes. Previously obtained mosquito RNA was used to synthesize cDNA using the iScript™ cDNA Synthesis Kit (Bio-Rad, CA, USA) and cDNA was used to amplify fragments of interest, using iProof™ High-Fidelity DNA Polymerase (BioRad, CA, USA) and specific primers containing T7 promoter sequences at the 5′-end (see Supplementary Table S3). PCR reactions of 50 µL included: 1× iProof HF Buffer, 10 mM of dNTP mix, 0.5 µM of each primer, 1 mM of MgCl2, 0.02 U/µL Proof DNA Polymerase and 50–500 ng of template cDNA. The conditions were: initial denaturation performed at 98 °C for 3 minutes; following for denaturation at 98 °C for 10 seconds, annealing at specific temperature (see Supplementary Table S3) for 30 seconds and extension at 72 °C for 15 seconds cycling for 35 cycles; and then a final extension at 72 °C for 10 minutes. Amplification results were analyzed on a 0.5× TBE, 1.2% (w/v) agarose gel, purified using Zigmoclean™ Gel DNA Recovery Kit (Zymo Research, USA) and sequenced. dsRNA was synthetized using the MEGAscript T7 kit (Ambion, Austin, TX, USA) according to manufacturer’s instructions and analyzed by spectrometry and agarose gel.

For dsRNA injection three independent assays per group (target gene) were conducted one week apart. For each group, 100 *P. berghei*-infected female were used to knockdown *prestinA*, *NDAE1* and both genes simultaneously. Similarly, the control groups were injected with an exogenous gene, mouse beta-2-microglobulin dsRNA (ds*β2 M*) (GenBank: NM_009735). Fourteen days PBM, cold anesthetized female mosquitoes were injected intrathoraxically with 69 nl (4 mg/ml) of each dsRNA using a nano-injector (Nanoject, Drummond Scientific, Broomall, PA, USA). For double knockdown a mixture of equal parts of dsRNA was used. At this time point, the majority of oocysts ruptured and the sporozoites start to invade salivary glands.

### Gene knockdown assessment

After dsRNA injection, the number of viable mosquitoes was evaluated using Log Rank, Breslow and Tarone-Ware tests^71, 72^. Four days post injection, three biological replicas containing 15 to 30 SGs per treatment were used to perform sporozoite quantification, by light microscopy using a haemocytometer^5^. Infection levels were compared between control and treated groups using the non-parametric Mann-Whitney test (SPSS v24.0)^73^. To assess gene knockdown efficiency three biological replicas of 20–40 SGs per group were used to extract total RNA and synthetize cDNA as described previously. qPCR assays were performed under the conditions aforementioned. Gene expression was analysed by the CFX Manager™ Software (Bio-Rad, CA, USA) using Pfaff method^63^.

## Electronic supplementary material

supplementary information
