# Supplementary material for: Solute carriers affect Anopheles stephensi survival and Plasmodium berghei infection in the salivary glands
Source: Sci Rep. 2017 Jul 21;7:6141. doi: 10.1038/s41598-017-06317-6 (PMC5522484; doi:10.1038/s41598-017-06317-6)
Supplement: Supplementary file 1 — supplementary information [file 41598_2017_6317_MOESM1_ESM.pdf]

Solute carriers affect *Anopheles stephensi* survival and *Plasmodium berghei* infection in the salivary glands

Couto, J., MSc[1,+]; Antunes, S., PhD [1,2,+], Pinheiro-Silva, R, PhD[1,+]; do Rosário V., PhD[2,+], de la Fuente J., PhD[3,4,+], Domingos, A., PhD[1,2,*,+]

[1]Instituto de Higiene e Medicina Tropical, Universidade Nova de Lisboa (GHMT-IHMT-UNL), Rua da Junqueira 100, 1349-008, Lisboa, Portugal

[2]Global Health and Tropical Medicine - Instituto de Higiene e Medicina, Universidade Nova de Lisboa (GHMT-IHMT-UNL), Rua da Junqueira 100, 1349-008 Lisboa, Portugal

[3]SaBio. Instituto de Investigación en Recursos Cinegéticos IREC, CSIC-UCLM-JCCM, Ronda de Toledo s/n, 13005 Ciudad Real, Spain

[4]Department of Veterinary Pathobiology, Center for Veterinary Health Sciences, Oklahoma State University, Stillwater, OK 74078, USA

[*]Corresponding author e-mail: adomingos@ihm.unl.pt

[+] These authors contributed equally to this work

Supplementary Table S1 - Validation of sialotranscriptomic results. Comparison of RNAseq and qPCR results from the selected 14 genes. Green = upregulated, black = no difference, red = downregulated. An *Anopheles gambiae ribosomal protein S7* orthologue was used as a reference gene.

**Gene name**	***Anopheles gambiae* gene**	***Anopheles stephensi* gene**	**RNA-seq**	**qPCR**
Histidine triad nucleotide binding protein 1	AGAP007702	ASTE001733		
arrestin domain containing 2	AGAP002691	ASTE009422		
paxillin	AGAP008532	ASTE007758		
-	AGAP009577	ASTE010763		
Class b basic helix-loop-helix protein	AGAP003844	ASTE011434		
chondroitin 4-sulfotransferase	AGAP005721	ASTE007038		
pebble	AGAP005796	ASTE009773		
Histidine triad nucleotide binding protein	AGAP006353	ASTE000811		
-	AGAP010794	ASTE008166		
serine protease inhibitor (serpin) 6	AGAP009212	ASTE001475		
prestin	AGAP010725	ASTE009391		
Solute carrier family 4 member	AGAP009736	ASTE003102		
ATP-dependent RNA helicase DDX24/MAK5	AGAP010702	ASTE010887		
Class B Scavenger Receptor 6 (CD36 domain)	AGAP004643	ASTE000417		

Up-regulated Down-regulated

Supplementary Fig. S1 - Pearson correlation coefficient of differential expression ratios between RNA-seq and qPCR. There was a strong correlation between the mRNA levels obtained by both RNA-seq and qPCR methods (Pearson's correlation coefficient = 0.667, P = 0.009).

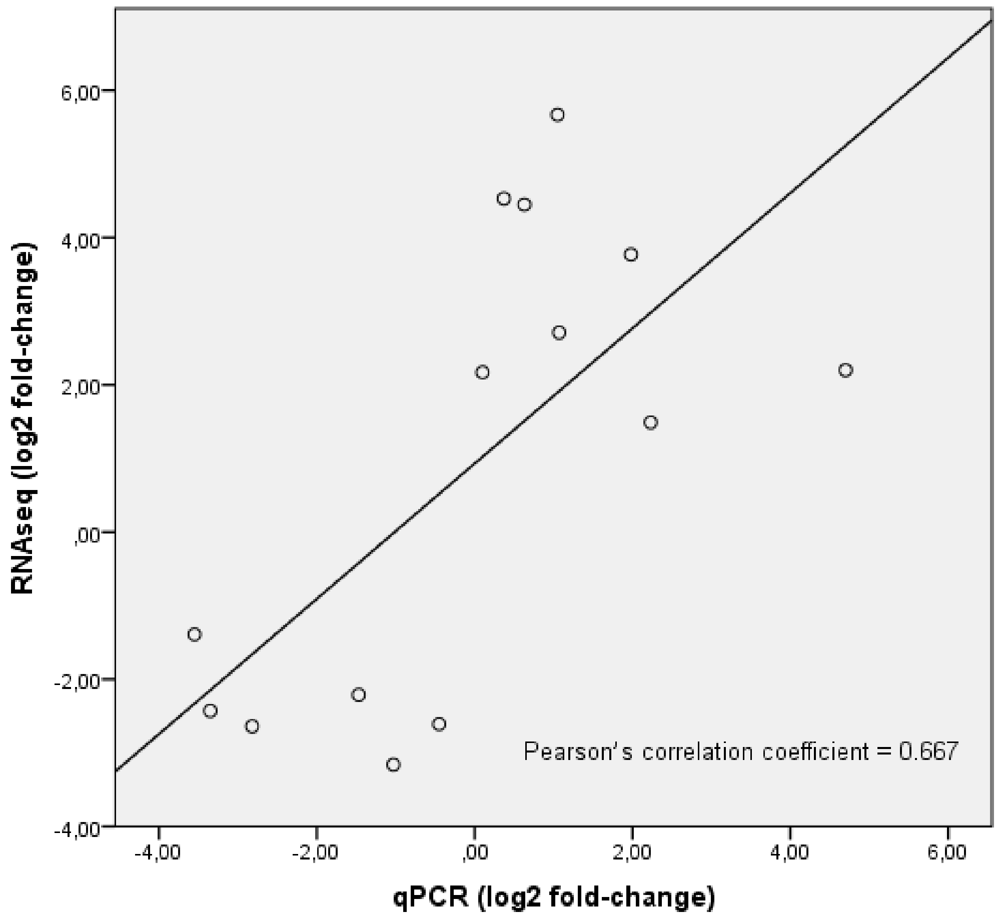

Supplementary Table S2 - List of primers used for validation by qPCR. The sequences of the primers designed for fourteen transcripts and respective conditions.

Gene	Vectorbase code	Primer Forward (5'-3')	Primer Reverse (5'-3')	Length (bp)	Annealing conditions (ºC/s)
SRPN6	**ASTE001475**	CAGCATGAACTGGAAGCGTA	GTTCGGGACATCGTCGTAGT	142	55
AGAP009577	**ASTE010763**	CATCCGGGTTCGATAGTACG	TGTTAGCACTGGGACGTCTG	105	56
Paxillin (PAX)	**ASTE007758**	GGTGTGAACACAACGCAGAA	GCAGTGGCTGCAGGTAAAGT	117	57
AGAP003844	**ASTE011434**	GAACAGTATCGGCTCGGGTA	TTGCAATGTTCCTTGAGGTG	116	55
AGAP005721	**ASTE007038**	CGCCAGCAGTTGCTACAGT	GCGCTGGTAGTGGTAGTGGT	132	58
AGAP005796	**ASTE009773**	GTTCCGGTTGGCAATCATAC	GTGCAGCTTGGTGAAGGTTT	103	55
AGAP007702	**ASTE001733**	TCAATCATCCGGACACGATA	CGAGACATCGTTGAAAGCAA	101	53
helicase DDX24/MAK5 (HELI)	**ASTE010887**	TTGAAACAAGATGCGACTGC	CGCTTAGTGATCCTGGCTTC	118	55
ARRDC2 (ARRE)	**ASTE009422**	GACAACCAAGGTCCAACGAT	GTACTGCACCCAACCGTACC	114	57
SCRB6	**ASTE000417**	CCTATCGCATGTCCACCTTT	ATCTTCATAGCCCCAGAGCA	113	55
AGAP006353	**ASTE000811**	CTCGTAATTCCTCGCAAACC	AGCCTTCCTCCAGTCCTTGT	124	56
AGAP010794	**ASTE008166**	TCGTACTTGCCCTTTCATCC	CAGTGAGGGCAATGATGTTG	120	54
AGAP01592 (RPS7)	**ASTE004816**	TCCTGGAGGATCTGGTGTTC	GATGGTGGTCTGCTGGTTCT	113	60
AGAP010725 (PRESTIN)	**ASTE009391**	ATTGCTGTCACGGTACCTTC	GGCTGAAATCTGGCAATGTT	101	59.2
AGAP009736 (NDAE1)	**ASTE003102**	TCTCGATCATTCTCGTCGTG	AAGCACGAGCAGTCCATCTT	193	60

Supplementary Table S3 - List of primers used for double-stranded RNA synthesis. RNAi-mediated gene-silencing assays were performed using specific primers containing T7 promoter sequences and annealing conditions.

Gene	Vectorbase code or GenBank code	Primer Forward (5'-3')*	Primer Reverse (5'-3')*	Length (bp)	Annealing conditions (ºC/s)
**beta-2 microglobulin (β2M)****	NM_009735	CACCCCCACTGAGACTGATACA	AATTAGGCCTCTTTGCTTTACCA	450	64
**ASTE009391 (Exon 1)*****	ASTE009391	GGAAGGGCATGAGAGTGGTA	AGTACACCAGCACCGGAAAG	471	64
**ASTE003102**	ASTE003102	CGTGAACGATCTGAAGCGTA	ATCGCGTAATGTAGCACACG	410	64

*All primers forward contained T7 promoter sequences (5' – TAATACGACTCACTATAGGGAGA – 3') at the 5'end.

**An exogenous gene, mouse beta-2microglobulin (*β2M*) (GenBank: NM_009735) was used as control for the knockdown experiments.

*** For ASTE009391 were tested three pairs of primers from exon 1, 5 and 6. But only primers from exon 1 amplified to high yields.

Supplementary Table S4 –Re-tested survival data from 16 to 18th day post blood meal. Kaplan-Meier statistics with values from the Log-Rank, Breslow, and Tarone-Ware tests. *P < 0.05, **P < 0.01, ***P < 0.001.

	**Gene knockdown**		
Re-tested data	*prestinA*	*NDAE1*	*prestinA + NDAE1*
**Log Rank (Mantel-Cox)**	0.790	17.697	73.367
***p* value**	0.374	<0.001***	<0.001***
**Breslow (Generalized Wilcoxon)**	3.763	25.348	38.351
***p* value**	0.052	<0.001***	<0.001***
**Tarone-Ware**	2.271	22.853	54.794
***p* value**	0.132	<0.001***	<0.001***
